# A Comparative Study between Conventional and Advanced Extraction Techniques: Pharmaceutical and Cosmetic Properties of Plant Extracts

**DOI:** 10.3390/molecules27072074

**Published:** 2022-03-23

**Authors:** Ezzouhra El Maaiden, Sarah Bouzroud, Boubker Nasser, Khadija Moustaid, Ayoub El Mouttaqi, Mohamed Ibourki, Hassan Boukcim, Abdelaziz Hirich, Lamfeddal Kouisni, Youssef El Kharrassi

**Affiliations:** 1 African Sustainable Agriculture Research Institute (ASARI), Mohammed VI Polytechnic University (UM6P), Laâyoune 70000, Morocco; ezzouhra.elmaaiden@um6p.ma (E.E.M.); sarah.bouzroud@um6p.ma (S.B.); ayoub.elmouttaqi@um6p.ma (A.E.M.); mohamed.ibourki@um6p.ma (M.I.); abdelaziz.hirich@um6p.ma (A.H.); lamfeddal.kouisni@um6p.ma (L.K.); 2 Laboratory of Biochemistry, Neurosciences, Natural Resources and Environment, Hassan First University of Settat, BP 577, Settat 26000, Morocco; boubker.nasser@uhp.ac.ma; 3 Laboratory of Applied Chemistry and Environment, Hassan First University of Settat, BP 577, Settat 26000, Morocco; khadija.moustaid@uhp.ac.ma; 4 AgroBioSciences Department (AgBS), Mohammed VI Polytechnic University (UM6P), Lot 660, Hay Moulay Rachid, Benguerir 43150, Morocco; hassan.boukcim@um6p.ma

**Keywords:** advanced extraction, conventional extraction, bioactive compounds, enzyme inhibition, oxidative stress, microwave-assisted extraction

## Abstract

This study aimed to compare the influence of extraction methods on the pharmaceutical and cosmetic properties of medicinal and aromatic plants (MAPs). For this purpose, the dried plant materials were extracted using advanced (microwave (MAE), ultrasonic (UAE), and homogenizer (HAE) assisted extractions) and conventional techniques (maceration, percolation, decoction, infusion, and Soxhlet). The tyrosinase, elastase, α-amylase, butyryl, and acetylcholinesterase inhibition were tested by using L-3,4 dihydroxy-phenylalanine, *N*-Succinyl-Ala-Ala-p-nitroanilide, butyryl, and acetylcholine as respective substrates. Antioxidant activities were studied by ABTS, DPPH, and FRAP. In terms of extraction yield, advanced extraction techniques showed the highest values (MAE > UAE > HAE). Chemical profiles were dependent on the phenolic compounds tested, whereas the antioxidant activities were always higher, mainly in infusion and decoction as a conventional technique. In relation to the pharmaceutical and cosmetic properties, the highest inhibitory activities against α-amylase and acetylcholinesterase were observed for Soxhlet and macerated extracts, whereas the highest activity against tyrosinase was obtained with MAE > maceration > Soxhlet. Elastase and butyrylcholinesterase inhibitory activities were in the order of Soxhlet > maceration > percolation, with no activities recorded for the other tested methods. In conclusion, advanced methods afford an extract with high yield, while conventional methods might be an adequate approach for minimal changes in the biological properties of the extract.

## 1. Introduction

Medicinal and aromatic plant (MAPs) extracts are currently attracting much attention because of their interesting phytochemical composition that has led to the development of new pharmacological and cosmetic drugs [1]. Extraction represents a critical step in the itinerary of phytochemical discovery from MAPs [2]. The final extract recovered can be substantially influenced by the type of extraction procedure used [2]. For a long time, extract preparation has been carried out using numerous ethnic groups, using maceration, percolation, infusion, and decoction methods. These extraction techniques have been reported to be conventional. Continuing to improve the extraction process, during the 18th century a progressive form of the decoction methods was reported, named Soxhlet [3]. However, these conventional methods, such as Soxhlet, have the disadvantage of requiring large solvent volumes and taking a long time to extract chemicals with poorer yields [4]. To face these challenges, research was oriented toward advanced, non-conventional extraction techniques. These include microwave-assisted, ultrasonic-assisted, and supercritical fluid extractions. Moreover, these advanced techniques were reported to possibly have a lower extract quality [4,5,6].

A comparison of the inhibitory activities of the conventional and advanced extraction techniques on the strategic enzymes targeted for the management of neurodegenerative disease, diabetes, and skin hyperpigmentation complications of different aromatic and medicinal plants has not been studied elsewhere. The broad objectives of this work were to compare conventional and advanced extraction techniques for the improvement of extract yields and the recovery of pharmaceutical and cosmetic properties of extracts obtained from aromatic and medicinal plants. The plants used in this study were: *Corrigiola litoralis* (L.), *Tamarix aphylla* (L. H.), *Pistacia lentiscus* (L.), *Cyperus rotundus* (L.), *Nardostachys jatamansi* (D. Don, DC.), and *Lavandula coronopifolia* Poir. In this sense, plant materials were extracted using advanced techniques, such as microwave- (MAE), ultrasonic- (UAE), and homogenizer-assisted (HAE) extractions, and conventional techniques such as maceration (MC), percolation (PR), decoction (DC), infusion (IF), and Soxhlet (SE). Pharmacological and cosmetic evaluations of different extracts were performed by screening the antioxidant activities and inhibitory potential against key enzymes of hyperpigmentation (tyrosinase), skin aging (elastase), diabetes (α-amylase), and neurodegenerative disease (acetylcholinesterase (AchE) and butyrylcholinesterase (BchE)).

## 2. Results and Discussion

### 2.1. Phytochemical Analysis

**Figure 1:**
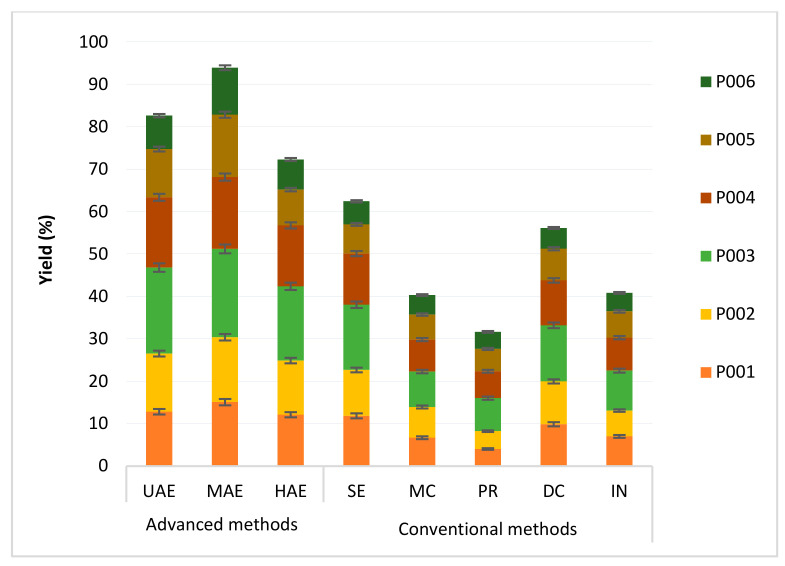
Extraction yields in the studied MAPs with advanced extraction techniques (microwave-, ultrasonic-, and homogenizer-assisted extractions) and conventional techniques (maceration, percolation, decoction, infusion, and Soxhlet). (P001) *Corrigiola litoralis* (L.), (P002) *Tamarix aphylla* (L. H.), (P003) *Pistacia lentiscus* (L.), (P004) *Cyperus rotundus* (L.), (P005) *Nardostachys jatamansi* (D. Don, DC.), and (P006) *Lavandula coronopifolia* Poir.

MAPs represent an important source of bio-compounds and serve as a starting material for cosmetic and pharmaceutics drugs’ biosynthesis. Extraction of these molecules is the first step for drug discovery. Further, these bio-compounds are present in low concentrations in plant materials. Regarding extraction solvents, a previous study demonstrated that absolute organic solvent afforded the lowest extraction efficiencies compared to aqueous and organic solvents [7]. Furthermore, transparent solvents such as dichloro-methane and hexane may not be used in MAE due to their inability to heat up under microwave radiations [7]. Methanol and water have a high microwave-absorbing ability and can heat up [8]. In this work, a hydroalcoholic solvent (70% water: 30% methanol) was used to study the influence of conventional and advanced extraction techniques on yield, pharmaceutical, and cosmetic properties of plant extracts. For this purpose, the dried plant materials were extracted using advanced (MAE, UAE, and HAE) and conventional techniques (MC, PR, DC, IN, and SE). Figure 1 shows extraction yields in selected MAPs using different extraction techniques.

As presented in Figure 1, the extraction yield ranged from 4% (P006-PR) to 20.8% (P003-MAE). Considering the techniques of extraction, extraction yields decreased in the following manner: MAE > UAE > HAE > SE> DC > IN ≥ MC > PR, for all plant species tested. The order of extraction yield with respect to the plant species used was: P003 > P004 > P002 > P001 > P005 > P006, for all extraction methods investigated (Figure 1). From these results, it is evident that the extraction yield of advanced methods (MAE, UAE, and HAE) is superior to conventional methods. For MAE, the direct heat kinetics affects the cellular wall of the plant, producing a high diffusion and increasing the partition rate of the solute into the solid matrix [9,10,11]. In the case of UAE and HAE, it can also be attributed to the effect of cavitation induced by the high ultrasound intensity [12].

Polyphenolic compounds as secondary metabolites are known to possess numerous pharmacological and cosmetics activities due to their ability to neutralize free radical species [13]. Therefore, the evaluation of the polyphenolic composition of extracts obtained using different extraction methods is critical. Here, total phenolic (TPC), flavonoid (TFC), and monomeric anthocyanin’s (TMAC) contents were evaluated using Folin–Ciocalteu, aluminum chloride, and pH differential methods, respectively (Table 1).

As shown in Table 1, TPC ranged from 5.08 to 88.3 mg GAE/g extract (for P002-MAE and P006-UAE), TFC ranged from 2.44 to 36.54 mg QE/g extract (for P002-DC and P004-UAE), and TAMC ranged from 0.36 to 6.36 mg C3G/g extract (for P002-HAE and P006-IN). P006 and P004 showed the highest polyphenolic contents compared to other plant species tested. The extraction method also affected the content of polyphenolic compounds extracted. UAE (non-conventional method) presented the best TPC and TFC contents, whereas IN (conventional method) presented the highest TMAC. The same results were presented in [14], where it was reported that UAE is an effective method for phenolic and flavonoid extraction. Jin et al. [15] compared the influence of extraction methods (UAE and maceration) on the polyphenolic contents (luteolin, flavonoid, and orientoside) and showed that maceration affords the lowest values [15]. In the same context, a study on the extraction of bio-compounds from the fruit of *Arbutus unedo* (L.) using MAE methods showed that maceration was more effective than MAE [16]. A study by Đurović et al. [17] revealed the effect of extraction techniques (maceration heat-assisted extraction and UAE) on the polyphenol content from serbyli herbs [17]. UAE produced high phenolic and flavonoid contents compared to other techniques tested. The effective ability of phenolics and flavonoids’ extraction by UAE was explained by sound waves used during the process of cavitation [18]. Moreover, a general consideration should be made considering MAE, which provided the highest yield (10% more than UAE) and lowest TPC. This could be attributed to the fact that direct kinetics and pressure effects can increase the solubility and diffusion of the solvent. However, this may cause solvent loss, leading to the decomposition of thermolabile components [19].

### 2.2. Antioxidant Properties

Antioxidants are the ability of compounds to inhibit the progression of oxidation stress induced by the imbalance between the production of reactive oxygen species (ROS) and their suppression. Antioxidant properties of studied extracts were tested using different assays (ABTS, DPPH, and FRAP). ABTS and DPPH assays are widely used techniques to test the capacity of plant extracts for scavenging 2,2-azinobis (3 ethylbenzothiazoline-6-sulfonicacid) and 2,2diphenyl-1-picrylh-ydrazyl, respectively [20,21,22]. FRAP activity is based on the potential of the plant extract to reduce ferric-tripyridyl triazine complex to the colored ferrous form. Data relating to radical scavenging and metal chelating assays are presented as equivalents of Trolox (Table 2).

**Figure 2:**
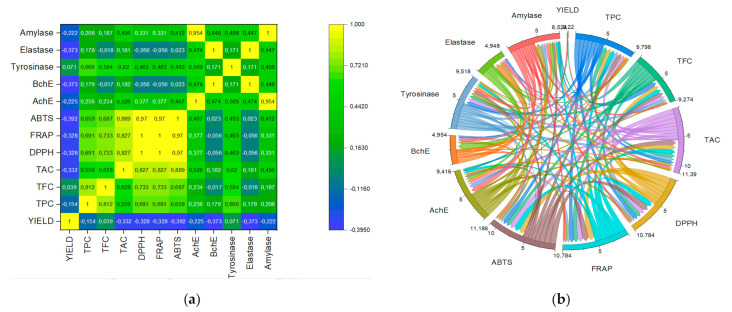
(**a**) Pearson’s correlation coefficient (R) and (**b**) chord diagram presenting the correlation between polyphenolics and biological properties. AchE and BchE are acetylcholinesterase and butyrylcholinesterase inhibition activities, respectively.

In Table 2, all tested extracts displayed interesting antioxidant activities carried out by ABTS, DPPH, and FRAP. Concerning plant species tested, we observed that antioxidant activities were influenced by the extraction methods and antioxidant tests used. P006 and P004 showed the highest antioxidant properties (DPPH, ABTS, and FRAP). Indeed, infusion and decoction as conventional extraction methods exhibited the highest antioxidant properties for all plant species tested. Whereas UAE, percolation, and maceration provided moderate activities and MAE afforded the lowest antioxidant activities for all plant species tested and using different antioxidant tests, such as DPPH, ABTS, and FRAP. This result corroborates with the study of Li et al. [23], who reported that decoction was more effective in the case of antioxidant extraction from various plant species compared to other extraction methods. However, Zhang et al. [24] declared that the limitation of this technique is that it does not afford the highest yields and has a vast number of waste-soluble impurities, and it is not suitable for the extraction of volatile compounds [24]. Our finding can be explained by the fact that the polyphenolic compounds (TPC, TMAC, and TMAC) tested in this study are not the only contributors to the antioxidant activities since other bio-compounds (alkaloids, tocopherols, carotenoids, and terpenes) can also contribute to the radical scavenging property [22]. Data analyses using Pearson’s correlation coefficient (R) and the chord diagram are presented in Figure 2.

Figure 2 shows that all polyphenolic compounds tested (TPC, TFC, TAAC) were positively correlated with the antioxidant property (R > 6) compared to other enzymatic assays tested, which showed lower correlation coefficients (R ≤ 5). It can be said that polyphenolic compounds such as TPC, TFC, and TAAC have a strange effect on the antioxidant activities, more so than other enzymatic assays tested. However, a negative correlation was observed between yield and other parameters tested.

### 2.3. Enzyme Inhibition Properties

**Figure 3:**
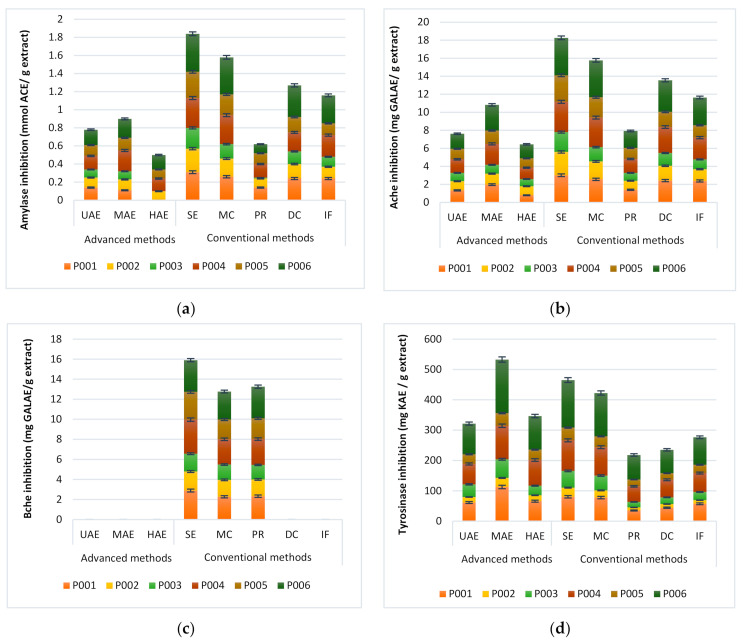

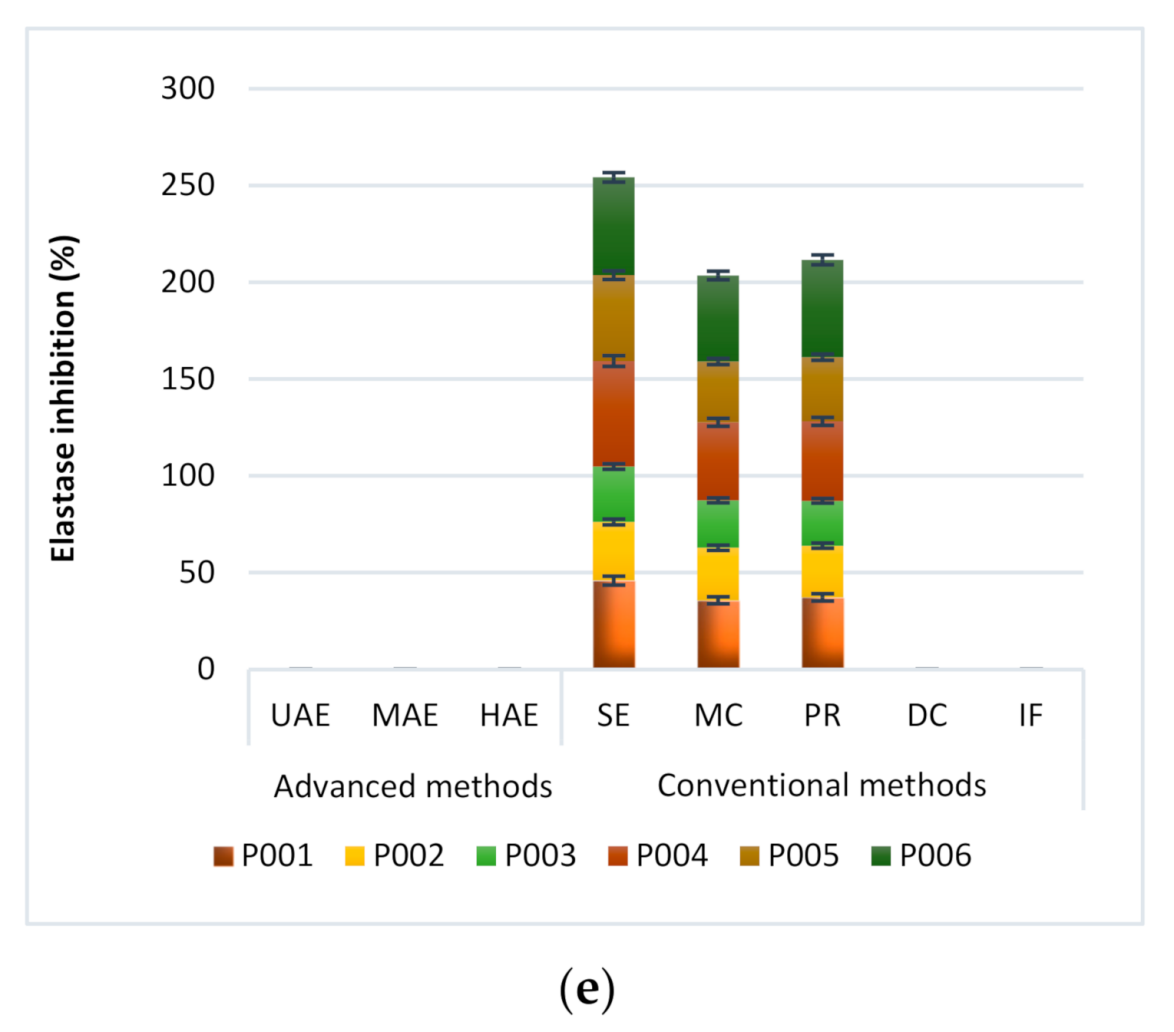
Enzyme inhibitory activities of MAP extracts obtained using conventional and non-conventional extraction techniques. Values are reported as mean ± standard deviation. The final concentration of tested extracts was 1 mg/mL. (**a**) α-amylase inhibition ability is presented as mmol acarbose equivalent per gram of MAPs extract (mmol ACE/g extract). (**b**) Acetylcholinesterase and (**c**) butyrylcholinesterase inhibitory activities are reported as milligrams galantamine equivalents per gram of MAPs extracts (mg GALAE/g extract). (**d**) Tyrosinase enzyme inhibitory is presented as kojic acid equivalent per gram of MAPs extracts (mg KAE/g extract). (**e**) Elastase enzyme inhibitory activity is reported in percentage (%). (P001) *Corrigiola litoralis* (L.), (P002) *Tamarix aphylla* (L. H.), (P003) *Pistacia lentiscus* (L.), (P004) *Cyperus rotundus* (L.), (P005) *Nardostachys jatamansi* (D. Don, DC.), and (P006) *Lavandula coronopifolia* Poir.

A comparison between the inhibition abilities of plant extracts obtained using the conventional and non-conventional extraction methods on the strategic enzymes implicated for management of neurodegenerative disease, diabetes, and skin hyperpigmentation complications of MAPs has not been reported elsewhere. For this purpose, MAP extracts generated by conventional and non-conventional extraction techniques were screened for their ability to inhibit tyrosinase, elastase, α-amylase, acetylcholinesterase, and butyrylcholinesterase using L-3,4-dihydroxy-phenylalanine, N-Succinyl-Ala-p-nitroanilide, acetylcholine, and butyrylcholine as respective substrates. The results are summarized in Figure 3.

Diabetes mellitus represents one of the main global diseases. Alpha-amylase (α-amylase) is a digestive enzyme responsible for breaking oligo/disaccharides into monosaccharides and delaying the absorbance of carbohydrates by inhibiting the enzyme responsible for carbohydrate hydrolysis. This leads to the optimization of postprandial blood glucose levels and may contribute to the treatment of diabetes and obesity. Natural α-amylase inhibitors from plant extracts represent an attractive approach for hyperglycemia bio-treatment [25,26,27,28,29,30]. Here, we tested the α-amylase inhibition ability of plant extracts obtained from 6 MAPs extracted using different conventional and non-conventional methods. α-amylase inhibitory activity was presented as mmol acarbose equivalent per gram of MAPs extract (mmol ACE/g extract). As a result, values ranged from 0 to 0.424 mmol ACE/g extract (for P001-HAE, P003-HAE, and P006-SE, respectively). P006 extracts presented the highest α-amylase inhibition activity compared to other plant species’ extracts for all extraction methods used. In terms of extraction method, the use of conventional methods of extraction (SE > MC > DC > IN) resulted in a better α-amylase inhibition activity in comparison with advanced methods (Figure 3a).

Cholinesterase such as AchE (acetylcholinesterase) and BchE (butyrylcholinesterase) are two key enzymes responsible of the breakdown of the neurotransmitter’s choline (acetyl and butyryl). In the management of some pathological cases such as Alzheimer’s, activities of these enzymes should be inhibited to cope with the non-compatible conditions. Inhibition of these enzymes with plant extracts was studied by many authors [31,32,33,34,35,36,37]. In our study, extracts obtained using conventional and non-conventional methods from different MAPs were tested for their potential enzyme inhibitory activities against AchE and BchE. The results were summarized in Figure 3b. Our data showed that conventional methods such as Soxhlet, maceration, and decoction afforded the best acetylcholinesterase (AchE) inhibition activity compared to non-conventional methods, whereas there was no inhibition activity against BchE decoction, infusion, UAE, MAE, and HAE extracts (Figure 3c).

Extracellular matrixes (ECM) represent the infrastructure of the skin foundation and consist of numerous components, such as collagen, elastin, and microfibrils. During the maturation process, this component undergoes a transformation process characterized by dryness, wrinkling, and skin laxity [38,39]. Melanin represents a major component of the skin, occurring as black or brown pigment synthesized by the melanogenesis process, and reinforces the protective role of skin as a physical barrier against environmental factors. However, an overproduction of melanin can cause a skin disorder. Tyrosinase represents the key enzyme of melanin production by transforming tyrosine to DOPA, which affords dopamine rather than melanin. Elastin is a key ECM protein that mediates skin tissue elasticity and connectivity. This protein can be degraded by the extracellular matrix enzyme, which is elastase, a serine proteinase. Consequently, the inhibition of skin-related enzymes, such as tyrosinase and elastase, is a critical approach for providing good skin integrity and youthfulness. In this work, the inhibitory effect of MAP extracts prepared using different extraction techniques against elastase and tyrosinase was summarized in Figure 3d,e. The summarized results show that P006 presented the highest tyrosinase and elastase inhibitory activities. In terms of the extraction technique used, we observed that the highest anti-elastase activity was recorded with conventional methods such as Soxhlet and maceration, and these methods presented good activity against tyrosinase. Like anti-BchE, anti-elastase activities were not found in the extracts obtained by decoction, infusion, UAE, MAE, and HAE.

Polyphenolic compounds have a great potential to protect against oxidative stress by prompting ROS scavenging. These natural compounds from plants with their antioxidant activities facilitate skin protection by controlling the activities of tyrosinase and elastase. In addition, they can alleviate disorders associated with diabetes, such as neurodegeneration [31,32,33,34,35,36,37]. In our study, P006 and P004 were found to be the best plant extracts for all tested parameters. P006 and P004 displayed specific adaptation mechanisms that naturally survive in the saline environment and hostile conditions and presented polyphenols such as phenolic, flavonoid, carotenoids, and vitamins that should be explored for medicine and cosmetic development [40,41,42]. Moreover, UAE, as a non-conventional extraction technique, afforded the highest phenolic compounds, whereas the cosmetics and pharmacological activities were less than with Soxhlet and maceration. These contrasting results of UAE can be explained by the sound wave used during the process of cavitation, the penetration of solvents into the matrix of the sample, and the extraction of bioactive compounds such as TPC and TFC in high quantities. However, these processes can affect the quality of bioactive compounds and induce their degradation and deactivation [28,42]. In the same regard, some studies evaluated the effect of extraction methods in the degradation of polyphenols and found that the highest energy type generated by the sonication mechanism and/or the highest temperature in a short time produced a decrease in the decomposition of these bio-compounds [19,24,42].

## 3. Materials and Methods

### 3.1. Collection and Identification of MAPs

Plant specimens were collected in 2020 and 2021 through fieldwork and standard collection practices [43,44,45]. Plant specimens were prepared and placed at the Herbarium of ASARI, Um6p Morocco. The Voucher specimen numbers, plant identification, and scientific name assignment are listed in Table 3 after cross-checking with the plant list (http://www.theplantlist.org/, accessed on 11 January 2022) and according to the best ethnopharmacology practices [46].

### 3.2. Extractions

Collected MAP samples were ground after being dried in the shade. Briefly, the powdered MAP samples (5 g) were extracted with 100 mL of methanol/water (30:70) with advanced and conventional techniques. Extraction is described in detail in Table 4. Extracts were filtrated and concentrated using a rotary evaporator (50 °C). Individual crude extract samples were labeled according to their extraction methods and a collection number ranging from P001 to P006 was assigned to the MAP studies. After being weighted for the extraction yield calculation, crude extracts were dissolved in DMSO at 1 mg/mL and stored at −20 °C until use.

### 3.3. Polyphenols Contents

Total phenolic content (TPC) was evaluated using the protocol of Vlase et al. [53]. Briefly, 0.250 mL of MAP extract (1 mg/mL) was incubated with 1 mL of Folin–Ciocalteu reagent (1:9 *v*/*v*) for 3 min, then re-incubated for 2 h after the addition of 0.750 mL of sodium carbonate (1%). The absorbance was noted at 760 nm. TPC was reported as milligram gallic acid equivalent per gram of MAP extract (mg GAE/g extract). Total flavonoid content (TFC) was measured according to the AlCl3 (aluminum chloride) protocol. Then, 1 mg/mL of MAP extract was mixed with AlCl3 (2%) and incubated for 10 min at lab temperature. The absorbance was measured at 415 nm. TFC was reported as milligram of quercetin equivalent per gram of extract (mg QE/g extract) [54]. Total monomeric anthocyanin’s content (TMAC) was evaluated by the differential pH method based on two buffer systems: potassium chloride buffer (pH 1.0, 0.25 mmol/L) and sodium acetate buffer (pH 4.5, 0.4 mol/L) [13]. Then, 0.250 mL of each MAP extract was added to 2 mL of each buffer system, with absorbance being noted from white at 510 nm and at 700 nm 15 s later. TMAC was reported as milligram of cyanidine-3-glucoside per gram of extract (mg C3G/g extract) [55].

### 3.4. Antioxidant Activities

Antioxidant activities were evaluated by ABTS, DPPH, and FRAP. For the DPPH radical scavenging assay, 0.4 mL of MAP extract (1 mg/mL) was mixed with 2 mL of DPPH methanolic solution (0.2 mm), incubated for 30 min, then measured by absorbance at 517 nm. DPPH activities were reported as Trolox equivalent (mg TE/g extract) [50]. In ABTS activities, by mixing in an equal amount of ABTS (7 mm) solution and potassium persulfate (2.45 mm) and allowing to react in the dark for 12 h, a green-colored ABTS+ radical was produced. Then, 1 mL of the resultant solution was diluted with 50 mL of methanol until an absorbance (0.7 ± 0.01) was obtained at 734 nm. Thereafter, 1 mL of MAP extract (1 mg/mL) was mixed with the ABTS+ solution. After 7 min, the absorbance was measured at 734 nm. The ABTS activity was reported as millimole of Trolox equivalents (mmol TE/g extract) [50]. For the FRAP activity, 200 µL of MAP extract was homogenized with 500 µL of potassium ferricyanide (1%), then incubated for 20 min (50 °C). Then, 2.5 mL of trichloroacetic acid (10%) was added to the solution and centrifugated (2200× *g*) for 20 min. Next, 500 µL of the upper layer of solution was mixed with 0.1 mL of ferric chloride and 0.5 mL of distilled water. The absorbance was measured at 700 nm and FRAP was reported as Trolox equivalent (mg TE/g extract) [56].

### 3.5. Enzymatic Inhibition Activities

To evaluate pharmaceutical and cosmetic property extracts, the inhibition of key enzymes of hyperpigmentation (tyrosinase), skin aging (elastase), diabetes (α-amylase), and neurodegenerative diseases (cholinesterase) was tested. The inhibition of tyrosinase was assessed using L-3,4-dihydroxyphenylalanine (L-DOPA) as a substrate according to [57]. In brief, into 96-well plates, 25 µL of MAP extract (1 mg/mL) was added to the tyrosinase sodium phosphate buffer (0.1 M, pH 6.8), 10 µL of tyrosinase in phosphate buffer (1500 units/mL), and 1.5 mL of tyrosinase (1.5 mm), and then incubated for 15 min. The reaction was initiated by adding 40 µL of L-DOPA (final concentration 2 mm) and activity was evaluated by following the increase in absorbance at 492 nm. The activity of elastase inhibition was detected by monitoring the liberation of *p*-Nitroanilide during the split of N-Succinyl-Ala-Ala-p-nitroanilide (AAAPVN) [58]. The elastase substrate (AAAPVN) mixture was prepared in Tris HCL solution (0.1232 M, pH 8.0) to obtain a concentration of 1.015 mm. Into the 96-well microplate sample solution, 10 µL of MAP extract was mixed with porcine pancreatic elastase (3.3 µg/mL) and incubated for 20 min. After that, the reaction mixture was initiated by adding the substrate and the inhibitory enzyme activity was measured at 410 nm using a microplate reader. Oleanolic acid served as a positive control (PC). For α-amylase inhibition ability, in 96-well microplates, 25 µL of MAP extract (1 mg/mL) was added to 50 µL of the α-amylase solution (240 U/mL), which was prepared in phosphate buffer (pH 6.9 with 6 mm sodium chloride), then the microplate was incubated (10 min, 37 °C). To initiate the reaction, 50 µL of starch solution (0.05%) was added to the mixture and re-incubated (30 °C, 10 min). A mixture of HCL (25 µL, 1 M) and iodine potassium iodide solution (100 µL) was added to stop the reaction. Absorbance was read at 630 nm and α-amylase inhibition activity was reported as acarbose equivalent (mmol ACE/g extract) [59]. In vitro, anti-cholinesterase activities (AchE and BchE) were performed according to [60]. Briefly, 50 µL of sample solution (1 mg/mL) was mixed with 5,5-dithio-bis(2-nitrobenzoic) acid and 25 µL of AchE or BchE solutions into the 96-well microplate, then incubated for 15 min (25 °C). The reaction was initiated by the addition of 25 µL of acetylthiocholine iodide or butyrylthiocholine chloride for AchE and BchE, then incubated for 10 min at 25 °C (final concentrations of 1.5 mm for acetylthiocholine iodide, or 4 mm for butyrylthiocholine iodide). Absorbance was measured at 405 nm. In vitro, acetylcholinesterase and butyrylcholinesterase inhibitory activities were reported as milligrams galantamine equivalents (mg GALAE/g extract).

### 3.6. Statistical Analysis

Data were reported as mean ± SD (*n* = 3). Data analysis was carried out using Microsoft excel 2010, Origin 8.5 (Origin Lab, Northampton, MA, USA), and SPSS 20.0 (IBM, Armonk, NY, USA) software. ANOVA and post-hoc Bonferroni were considered statistically significant if *p* < 0.05.

## 4. Conclusions

This study has shown the influence of conventional and advanced extraction techniques on the yield, phytochemical, and biological activities of MAP extracts. According to the obtained results, advanced extraction methods (microwave-, ultrasonic-, and homogenizer-assisted) present a heating and sonication mechanism that significantly reduces the time of extraction (from a few seconds to half an hour). These advanced methods of extraction presented higher extraction yields compared to conventional methods (maceration, percolation, decoction, infusion, and Soxhlet). Meanwhile, Soxhlet, maceration, and percolation offered higher α-amylase, acetylcholinesterase, elastase, and butyrylcholinesterase levels. The highest activity against tyrosinase was obtained with MAE, followed by macerated and Soxhlet. In addition, this work appraised the possible application of *Lavandula coronopifolia* Poir for the management of key enzymes of hyperpigmentation (tyrosinase), skin aging (elastase), diabetes (α-amylase), and neurodegenerative diseases.

## Figures and Tables

**Table 1:** Total polyphenol contents in MAPs extracted with different extraction techniques.

		UAE	MAE	HAE	SE	MC	PR	DC	IF
P001	TPC	56.41 ± 0.01	17.70 ± 0.00	40.20 ± 0.00	30.71 ± 0.01	38.86 ± 0.06	33.00 ± 0.00	22.20 ± 0.08	28.77 ± 0.09
	TFC	22.55 ± 0.00	11.48 ± 0.00	15.56 ± 0.02	8.88 ± 0.00	13.2 ± 0.07	12.22 ± 0.04	7.09 ± 0.10	15.08 ± 0.09
	TAAC	1.60 ± 0.11	2.37 ± 0.01	1.28 ± 0	2.08 ± 0.02	2.71 ± 0.02	2.8 ± 0.04	2.22 ± 0.03	4.06 ± 0.01
P002	TPC	14.90 ± 0.00	5.08 ± 0.00	15.09 ± 0.00	9.06 ± 0.01	11.92 ± 0.05	9.77 ± 0.00	6.04 ± 0.04	5.99 ± 0.10
	TFC	7.14 ± 0.11	2.87 ± 0.00	5.72 ± 0.04	2.9 ± 0.00	4.68 ± 0.10	4.23 ± 0.03	2.44 ± 0.03	6.04 ± 0.01
	TAAC	0.43 ± 0.01	0.71 ± 0.02	0.36 ± 0.06	0.43 ± 0.01	0.91 ± 0.03	0.85 ± 0.01	0.64 ± 0.04	1.07 ± 0.01
P003	TPC	30.70 ± 0.00	8.99 ± 0.00	27.60 ± 0.00	20.90 ± 0.01	24.43 ± 0.09	15.77 ± 0.00	11.10 ± 0.09	13.47 ± 0.07
	TFC	12.28 ± 0.01	5.38 ± 0.00	9.77 ± 0.08	4.44 ± 0.00	5.31 ± 0.00	8.35 ± 0.06	3.60 ± 0.01	11.05 ± 0.08
	TAAC	0.79 ± 0.01	0.95 ± 0.03	0.65 ± 0.01	0.97 ± 0.02	1.98 ± 0.08	1.75 ± 0.01	1.50 ± 0.03	2.21 ± 0.00
P004	TPC	55.31 ± 0.01	25.66 ± 0.00	50.40 ± 0.00	33.61 ± 0.01	46.83 ± 0.05	42.30 ± 0.00	28.90 ± 0.01	31.21 ± 0.01
	TFC	36.54 ± 0.02	24.98 ± 0.00	32.44 ± 0.04	23.86 ± 0.00	29.11 ± 0.01	26.11 ± 0.02	22.32 ± 0.11	34.18 ± 0.02
	TAAC	2.08 ± 0.00	2.86 ± 0.03	1.84 ± 0.01	2.25 ± 0.00	3.60 ± 0.07	3.37 ± 0.07	2.42 ± 0.02	3.98 ± 0.00
P005	TPC	20.80 ± 0.00	11.30 ± 0.00	20.90 ± 0.00	15.89 ± 0.00	17.88 ± 0.00	17.09 ± 0.00	10.80 ± 0.02	13.67 ± 0.10
	TFC	8.32 ± 0.10	5.46 ± 0.00	7.15 ± 0.01	4.32 ± 0.00	5.84 ± 0.01	5.35 ± 0.04	4.52 ± 0.03	8.35 ± 0.04
	TAAC	0.77 ± 0.01	1.05 ± 0.02	0.82 ± 0.01	0.98 ± 0.01	1.5 ± 0.07	1.28 ± 0.02	0.99 ± 0.09	1.50 ± 0.01
P006	TPC	88.30 ± 0.01	40.40 ± 0.00	78.44 ± 0.00	50.60 ± 0.00	71.33 ± 0.11	55.40 ± 0.06	38.64 ± 0.11	45.33 ± 0.06
	TFC	35.32 ± 0.00	18.12 ± 0.00	28.53 ± 0.02	15.44 ± 0.00	22.15 ± 0.05	20.24 ± 0.05	15.15 ± 0.02	31.38 ± 0.01
	TAAC	2.77 ± 0.02	4 ± 0.11	2.73 ± 0.01	3.26 ± 0.03	5.56 ± 0.13	5.13 ± 0.03	3.64 ± 0.05	6.36 ± 0.01

Values are reported as mean ± standard deviation and were significant (*p* < 0.05). TPC was reported as milligrams gallic acid equivalents (mg GAE/g extract). TFC was reported as quercetin equivalent (MG QE/g extract). TMAC was reported in mg C3G/g extract. Microwave- (MAE), ultrasonic- (UAE), and homogenizer (HAE)-assisted extractions. MC: maceration, PR: percolation, DC: decoction, IN: infusion, and SE: Soxhlet extracts. (P001) *Corrigiola litoralis* (L.), (P002) *Tamarix aphylla* (L. H.), (P003) *Pistacia lentiscus* (L.), (P004) *Cyperus rotundus* (L.), (P005) *Nardostachys jatamansi* (D. Don, DC.), and (P006) *Lavandula coronopifolia* Poir.

**Table 2:** Antioxidant properties of MAP extracts obtained using different conventional and advanced extraction techniques.

		UAE	MAE	HAE	SE	MC	PR	DC	IF
P001	ABTS	128.94 ± 0.05	74.34 ± 0.05	120.54 ± 0.05	93.24 ± 0.04	163.37 ± 0.07	138.57 ± 0.1	168.81 ± 0.05	236.85 ± 0.07
	DPPH	111.14 ± 0.05	50.58 ± 0.01	82.00 ± 0.00	63.43 ± 0.04	87.71 ± 0.04	94.27 ± 0.07	114.85 ± 0.05	161.14 ± 0.04
	FRAP	177.82 ± 0.10	80.91 ± 0.02	131.19 ± 0.07	101.5 ± 0.07	140.35 ± 0.06	150.85 ± 0.06	183.77 ± 0.08	257.82 ± 0.1
P002	ABTS	38.05 ± 0.05	21.34 ± 0.04	25.16 ± 0.06	25.37 ± 0.04	49.97 ± 0.06	41.04 ± 0.01	63.38 ± 0.02	62.59 ± 0.03
	DPPH	34.00 ± 0.00	14.49 ± 0.05	17.11 ± 0.02	17.25 ± 0.06	25.88 ± 0.08	27.92 ± 0.05	43.11 ± 0.11	42.58 ± 0.02
	FRAP	54.40 ± 0.07	23.22 ± 0.03	27.37 ± 0.07	27.61 ± 0.02	41.4 ± 0.01	44.66 ± 0.01	68.9 ± 0.00	68.11 ± 0.01
P003	ABTS	87.78 ± 0.06	37.77 ± 0.02	56.50 ± 0.01	46.58 ± 0.01	102.48 ± 0.08	66.24 ± 0.04	115.92 ± 0.05	128.94 ± 0.03
	DPPH	69.71 ± 0.02	25.67 ± 0.07	38.42 ± 0.03	31.68 ± 0.08	59.71 ± 0.02	45.05 ± 0.06	78.85 ± 0.06	87.71 ± 0.02
	FRAP	111.54 ± 0.04	41.07 ± 0.11	61.47 ± 0.06	50.69 ± 0.02	95.55 ± 0.08	72.09 ± 0.00	126.17 ± 0.17	140.34 ± 0.04
P004	ABTS	141.12 ± 0.03	107.77 ± 0.07	131.05 ± 0.06	121.38 ± 0.03	196.56 ± 0.06	177.66 ± 0.06	211.68 ± 0.08	232.26 ± 0.07
	DPPH	133.71 ± 0.05	73.32 ± 0.02	89.14 ± 0.04	82.58 ± 0.09	96.00 ± 0.00	120.85 ± 0.06	144.00 ± 0.00	158.00 ± 0.00
	FRAP	213.94 ± 0.02	117.30 ± 0.08	142.62 ± 0.06	132.11 ± 0.04	153.60 ± 0.00	193.36 ± 0.06	230.4 ± 0.06	252.8 ± 0.00
P005	ABTS	66.73 ± 0.01	47.46 ± 0.07	57.41 ± 0.01	45.37 ± 0.02	75.10 ± 0.01	71.78 ± 0.02	87.76 ± 0.08	87.36 ± 0.03
	DPPH	51.08 ± 0.09	32.29 ± 0.02	39.05 ± 0.07	30.86 ± 0.06	45.40 ± 0.09	48.82 ± 0.02	59.71 ± 0.08	59.42 ± 0.02
	FRAP	81.73 ± 0.04	51.65 ± 0.02	62.50 ± 0.02	49.37 ± 0.01	72.64 ± 0.03	78.13 ± 0.03	95.55 ± 0.05	95.08 ± 0.08
P006	ABTS	212.52 ± 0.03	169.68 ± 0.09	190.26 ± 0.01	162.12 ± 0.02	299.58 ± 0.08	232.68 ± 0.08	329.44 ± 0.01	370.86 ± 0.06
	DPPH	203.81 ± 0.04	115.44 ± 0.10	129.43 ± 0.03	110.29 ± 0.09	144.57 ± 0.08	158.28 ± 0.09	224.12 ± 0.11	252.28 ± 0.01
	FRAP	326.07 ± 0.04	184.68 ± 0.01	207.09 ± 0.10	176.45 ± 0.02	231.32 ± 0.02	253.26 ± 0.03	358.50 ± 0.00	403.66 ± 0.06

Values are reported as mean ± standard deviation and are significant (*p* < 0.05). ABTS, FRAP, and DPPH were reported as Trolox equivalent per grams of extract (mg TE/g extract). Microwave- (MAE), ultrasonic- (UAE), and homogenizer (HAE)-assisted extractions. MC: maceration, PR: percolation, DC: decoction, IN: infusion, and SE: Soxhlet extracts. (P001) *Corrigiola litoralis* (L.), (P002) *Tamarix aphylla* (L. H.), (P003) *Pistacia lentiscus* (L.), (P004) *Cyperus rotundus* (L.), (P005) *Nardostachys jatamansi* (D. Don, DC.), and (P006) *Lavandula coronopifolia* Poir.

**Table 3:** Description of collected plant species investigated in this study.

Collection Number	Scientific Name	Vernacular Name	Family Name	Plant Part
P001	*Corrigiola litoralis* L.	Serghina	Caryophyllaceae	Roots
P002	*Tamarix aphylla* (L.) H.	Lyag	Tamaricaceae	Fruits
P003	*Pistacia lentiscus* L.	Derou	Anacardiaceae	Secondary branching
P004	*Cyperus rotundus* L.	Saâd	Cyperaceae	Seeds
P005	*Nardostachys jatamansi* (D. Don) DC.	Assanbal	Valerianaceae	Leaves
P006	*Lavandula coronopifolia* Poir.	Khailat lkheil	Lamiaceae	Whole plant

**Table 4:** Detailed explanation of the extraction procedures used in this study.

	Methods	Temperature	Time	Operating Protocol	References
Advanced Methods	Ultrasonic-assisted extraction (UAE)	60 °C	1 h	Ultrasonic bath (application power: 120W; frequency range: 50–60 Hz)	[47]
	Microwave-assisted extraction (MAE)	60 °C	15 min	Microwave Extraction System (300 W during 15 min; divided into 3 identical cycles of 5 min; 5 min between cycles of microwave power)	[48]
	Homogenizer-assisted extractions (HAE)	40 °C	5 min	Ultra-turrax (6000 g)	[49]
Conventional Methods	Soxhlet (SE)	≤50 °C	7 h	Classic Soxhlet Apparatus	[50]
	Maceration (MC)	Room temperature	24 h	Occasionally shaking	[51]
	Percolation (PR)	Room temperature	6 h	The movement and filtrate of methanol/water through plant materials	[52]
	Decoction (DC)	78 °C	30 min	Boiling	[51]
	Infusion (IF)	78 °C	30 min	Added boiling methanol/water to plant materials	[51]

## Data Availability

Not applicable.
